# Is Halcyon feasible for single thoracic or lumbar vertebral segment SBRT?

**DOI:** 10.1002/acm2.13458

**Published:** 2021-11-29

**Authors:** Fang Li, Jeonghoon Park, Ron Lalonde, Si Young Jang, Maria Stefania diMayorca, John C. Flickinger, Andrew Keller, Mohammed Saiful Huq

**Affiliations:** ^1^ Department of Radiation Oncology UPMC Hillman Cancer Center Pittsburgh Pennsylvania USA; ^2^ Department of Medical Physics Memorial Sloan Kettering Cancer Center Basking Ridge New Jersey USA; ^3^ Department of Radiation Oncology Boston Medical Center Boston Massachusetts USA

**Keywords:** Halcyon, spine metastasis, stereotactic body radiation therapy (SBRT)

## Abstract

**Purpose:**

Halcyon linear accelerators employ intensity‐modulated radiation therapy (IMRT) and stereotactic body radiation therapy (SBRT) techniques. The Halcyon offers translational, but not rotational, couch correction, which only allows a 3 degrees of freedom (3‐DOF) correction. In contrast, the TrueBeam (TB) linear accelerator offers full 6‐DOF corrections. This study aims to evaluate the difference in treatment plan quality for single thoracic or lumbar vertebral segment SBRT between the Halcyon and TB linear accelerators. In addition, this study will also investigate the effect of patient rotational setup errors on the final plan quality.

**Methods:**

We analyzed 20 patients with a single‐level spine metastasis located between the T7 and L5 vertebrae near the spinal canal. The median planning target volume was 52.0 cm^3^ (17.9–138.7 cm^3^). The median tumor diameter in the axial plane was 4.6 cm (range 1.7–6.8 cm), in the sagittal plane was 3.3 cm (range 2–5 cm). The prescription doses were either 12–16 Gy in 1 fraction or 18–24 Gy in 3 fractions. All patients were treated on the TB linear accelerator with a 2.5 mm Multi‐Leaf Collimator (MLC) leaf width. Treatment plans were retrospectively created for the Halcyon, which has a 5 mm effective MLC leaf width. The 20 patients had a total of 50 treatments. Analysis of the 50 cone beam computed tomography (CBCT) scans showed average rotational setup errors of 0.6°, 1.2°, and 0.8° in pitch, yaw, and roll, respectively. Rotational error in roll was not considered in this study, as the original TB plans used a coplanar volumetric modulated arc therapy (VMAT) technique, and each 1° of roll will contribute an error of 1/360. If a plan has 3 arcs, the contribution from errors in roll will be < 0.1%. To simulate different patient setup errors, for each patient, 12 CT image datasets were generated in Velocity AI with different rotational combinations at a pitch and yaw of 1°, 2°, and 3°, respectively. We recalculated both the TB and Halcyon plans on these rotated images.  The dosimetric plan quality was evaluated based on the percent tumor coverage, the Conformity Index (CI), Gradient Index (GI), Homogeneity index (HI), the maximum dose to the cord/cauda, and the volume of the cord/cauda receiving 8, 10, and 12 Gy (V8Gy, V10Gy and V12Gy). Paired *t*‐tests were performed between the original and rotated plans with a significance level of 0.05.

**Results:**

The Eclipse based VMAT plans on Halcyon achieved a similar target coverage (92.3 ± 3.0% vs. 92.4 ± 3.3%, *p* = 0.82) and CI (1.0 ± 0.1 vs. 1.1 ± 0.2, *p* = 0.12) compared to the TB plans. The Gradient index of Halcyon is higher (3.96 ±0.8) than TB (3.85 ±0.7), but not statistically significant. The maximum dose to the spinal cord/cauda was comparable (11.1 ± 2.8 Gy vs. 11.4 ± 3.6 Gy, *p* = 0.39), as were the V8Gy, V10Gy and V12Gy to the cord/cauda. The dosimetric influence of patient rotational setup error was statistically insignificant for rotations of up to 1° pitch/yaw (with similar target coverage, CI, max cord/cauda dose and V8Gy, V10Gy, V12Gy for cord/cauda). The total number of monitor units (MUs) for Halcyon (4998 ± 1688) was comparable to that of TB (5463 ± 2155) (*p *= 0.09).

**Conclusions:**

The Halcyon VMAT plans for a single thoracic or lumbar spine metastasis were dosimetrically comparable to the TB plans. Patient rotation within 1° in the pitch and yaw directions, if corrected by translation, resulted in insignificant dosimetric effects. The Halcyon linear accelerator is an acceptable alternative to TB for the treatment of single thoracic or lumbar spinal level metastasis, but users need to be cautious about the patient rotational setup error.  It is advisable to select patients appropriately, including only those with the thoracic or lumbar spine involvement and keeping at least 2 mm separation between the target and the cord/cauda. More margin is needed if the distance between the isocenter and cord/cauda is larger. It is advisable to place the planning isocenter close to the spinal canal to further mitigate the rotational error.

**Summary:**

We simulated various scenarios of patient setup errors with different rotational combinations of pitch and yaw with 1°, 2°, and 3°, respectively. Rotation was corrected with translation only to mimic the Halcyon treatment scenario. Using the Halcyon for treating a tumor in a single thoracic or lumbar vertebral segment is feasible, but caution should be noted in patients requiring rotational corrections of > 1° in the absence of 6‐DOF correction capabilities.

## 1 INTRODUCTION

Stereotactic body radiation therapy (SBRT) is a safe and effective treatment for spinal metastases, which has been utilized over the past few decades.^1^, ^2^ The major advantage of SBRT is that a higher dose per fraction (> 5 Gy/fraction) can be delivered in up to five fractions to yield a greater biological equivalent dose (BED) when compared to conventional radiation. Due to the proximity of spinal tumor targets to the radiation‐sensitive spinal cord/cauda, the tumor margin is typically small, and a steep dose gradient is necessary. In addition, spinal tumors often have a concave shape around the spinal canal. To deliver the highly conformal dose to the tumors while avoiding the spinal cord/cauda, patient positioning and immobilization become critical to ensure both adequate tumor coverage and spinal cord/cauda sparing.

SBRT requires adequate patient immobilization and positioning. With the introduction of three‐dimensional volumetric imaging (e.g., cone beam computed tomography (CBCT)) and customized body vacuum bags (BodyFIX^®^), patient set‐up accuracy can be achieved within 1 mm at the target isocenter during daily treatment.^3^, ^4^, ^5^, ^6^, ^7^ BodyFIX^®^ are commonly used in SBRT treatment to maximize repositioning accuracy and intra‐treatment patient stability by reducing both involuntary and voluntary patient movement. In addition to BodyFIX^®^, the treatment couch can compensate for a certain degree of positional errors to better reproduce the patient position at CT‐Simulation. Mancosu^8^ et al. analyzed 2945 CBCTs from brain, lung, liver, pancreas, and prostate treatments and found the maximum absolute values for pitch and roll rotations were 0.7°±0.7° and 0.8°±0.8° in brain, with the lowest values for rotation of 0.4°±0.5° and 0.5°±0.6° in pancreas. Therefore, they suggest that the 6 degrees of freedom (DOF) robotic couch should be used on brain tumors, if available.

However, not all clinics are equipped with a 6‐DOF couch needed for the CBCT shift. Often, only a translational correction is available in some machines, like Varian Halcyon (Varian Medical Systems, Palo Alto, CA). While the Varian TrueBeam (TB) STx linear accelerator offers the full 6‐DOF correction, the Halcyon machine's couch allows only translational but not rotational correction (i.e., only 3‐DOF correction). In addition, when proper target coverage and spinal cord/cauda sparing are of concern, the TB is preferable due to smaller leaf width of 2.5 mm (2.5 mm leaf width is an optional purchase). On the other hand, the Halcyon's MLC collimation system has qualities that makes it clinically desirable,^9^ despite the concerns of inferior plan quality from the 5 mm effective leaf width. Halcyon has a dual‐layer Multi Leaf Collimator (MLC) system. It has a high leaf speed capability of 5 cm/s, compared to 2.5 cm/s in TB, resulting in faster intensity‐modulated deliveries with less intra‐fraction motion and more efficient patient throughput.

As such, both physical and mathematical efforts have been made to compensate for pitch and roll rotation when a 6‐DOF robotic couch is not available. To correct for pitch and yaw setup errors, Boswell^10^ et al. proposed moving the couch at a very low velocity along the anterior‐posterior and left‐right axes during Tomotherapy treatment. Several studies have looked at mathematical beam manipulations instead of a physical couch correction. Yue^11^ et al. used three matrix transformations to adjust for shifts of target isocenter by calculating the necessary shifts in gantry position, couch position, and collimator angles of the beams. The transformed beams will then possess the same positions and orientations relative to the target at treatment as in the original plan. This technique was tested on a head phantom. They found that the target shift was fully corrected in treatment and found excellent agreement in target dose coverage between the plan and the treatment. Fu^12^, ^13^ et al. used the same matrix transformation method and reported no dosimetric impact from up to 3° in rotational setup errors in prostate IMRT. They reported that the dosimetric impact for head and neck IMRT cases depended on the proximity of the organ at risk (OAR) to the tumor and that the rotational setup error might need to be routinely corrected on a case‐by‐case basis. However, these techniques are still in the academic stage and are not yet clinically available.

Other studies^14^, ^15^, ^16^, ^17^ attempted to compensate for rotational errors with a different approach. The studies conducted by Kim^14^ et al., Oh^15^ et al., Guckenberger^16^ et al., and Li^17^ et al. were based on patient setup errors observed on daily CBCT imaging during treatment. Since the setup errors are different between individuals, the beams are ad hoc recalculated on CBCT for dose variations, and comparisons between individuals are limited due to different combinations of rotational correction.  A more systematic comparison of rotational treatment plans between individuals can be achieved by pooling together data with the same degree of rotation. Study by Peng^18^ et al. was the only study that simulated patient rotation as opposed to using the rotational errors seen on daily CBCT. This group studied 10 patients with brain tumors who received Intensity‐Modulated Radiotherapy (IMRT). They rotated the CT images and contours using their in‐house MATLAB script and then performed Dose Volume Histogram (DVH) analysis on the recalculated dose distribution. In most cases, 95% CTV coverage was maintained with rotation setup errors up to 3° for intracranial tumors with a 3 mm CTV‐to‐PTV margin expansion treated with IMRT. However, for large targets with irregular or elliptical shapes, the target coverage decreased significantly with rotational errors of 5° or more. The results indicate that, even in the absence of translational setup errors, setup margins are needed to account for rotational setup errors. This study did not use translation to correct for rotation, which is slightly different than the standard clinical Halcyon treatment.

A literature search did not show any systematic studies on how to correct patient rotational errors with translation only in spine SBRT treatment, and there is no published data on the magnitude of rotational setup errors we can mitigate with translational corrections alone without compromising the target coverage and exceeding OAR dose limits. This will be very helpful for current Halcyon treatments.

We performed a comprehensive retrospective case‐control study on the difference in SBRT plan quality between the Halcyon and Truebeam linear accelerators in the treatment of a single thoracic or lumbar vertebral segment metastasis. In addition, we investigated the dosimetric effect of patient rotational setup errors when only translational corrections are available.

## 2 METHODS AND MATERIALS

### 2.1 Patients and volumes

**FIGURE 1:**
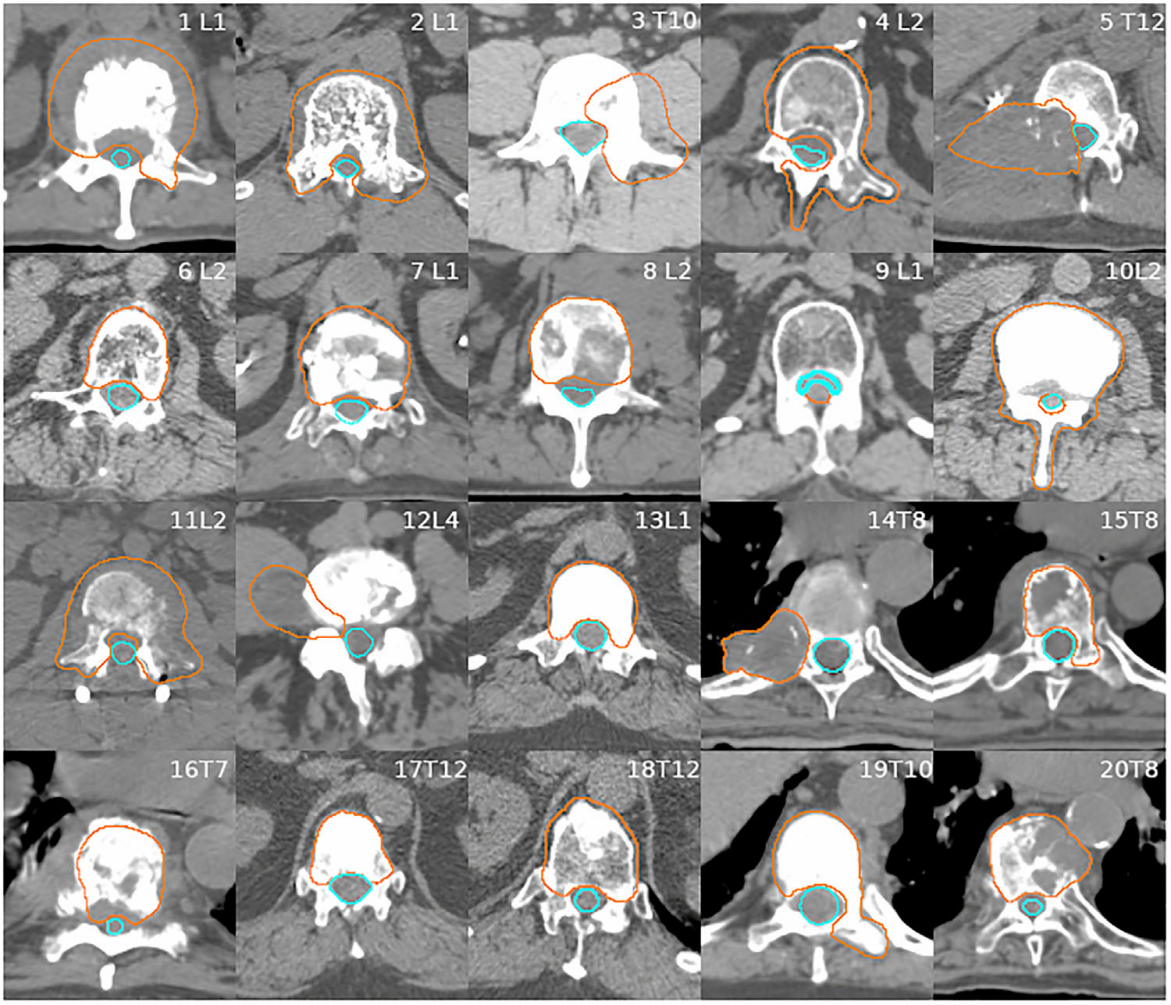
Axial view of 20 patients with PTV (orange) and cord (cyan) contoured

We analyzed a total of 20 patients (9 thoracic, 11 lumbar) with a single‐level spinal metastasis, located between T7 and L5 near the spinal canal (Figure 1).  All contouring followed RTOG‐0631 guidelines, and on doctor's clinical judgement. We intentionally omitted patients with cervical spine metastases, as the cervical spine has a lot of mobility, and these tumors are safer to treat with a 6‐DOF couch. These 20 patients were treated on a TB linear accelerator with a 6‐DOF perfect pitch couch and 2.5 mm high definition MLC (HD MLC) at our institution between January 2018 and July 2018.

Figure 1 displays the axial view at the target center and spinal cord or cauda equina for each patient. A 2 mm margin was added from gross tumor volume (GTV) to planning tumor volume (PTV). To account for the potential variations in patient position, the inherent imprecision of image fusion, and the physiological spinal cord/cauda motion and contouring uncertainty due to pixel size, the cord/cauda was either contoured with a 2 mm margin around it or as the entire spinal canal^19^, ^20^ (except Pt #5, #9, and #12, who have no margins around the cord/cauda).

The median tumor volume was 52.0 cm^3^ (17.9–138.7 cm^3^). The median tumor diameter in the superior‐inferior direction was 3.3 cm (2–5 cm) and 4.6 cm (1.7–6.8 cm) in left‐right direction. The prescription doses were 12–16 Gy in 1 fraction or 18–24 Gy in three fractions. We scaled all prescription doses to 16 Gy in one fraction in order to average the maximum cord/cauda dose from the plans. All patients were planned in Eclipse AAA V15.6 and treated in the TB linear accelerator with the coplanar volumetric‐modulated arc therapy (VMAT) technique. Their treatments were then retrospectively planned for evaluation on the Halcyon with VMAT technique using the same set of planning CT images and contours. No patients were treated on the Halcyon.

### 2.2 Simulation of patient rotation with respect to isocenter

**FIGURE 2:**
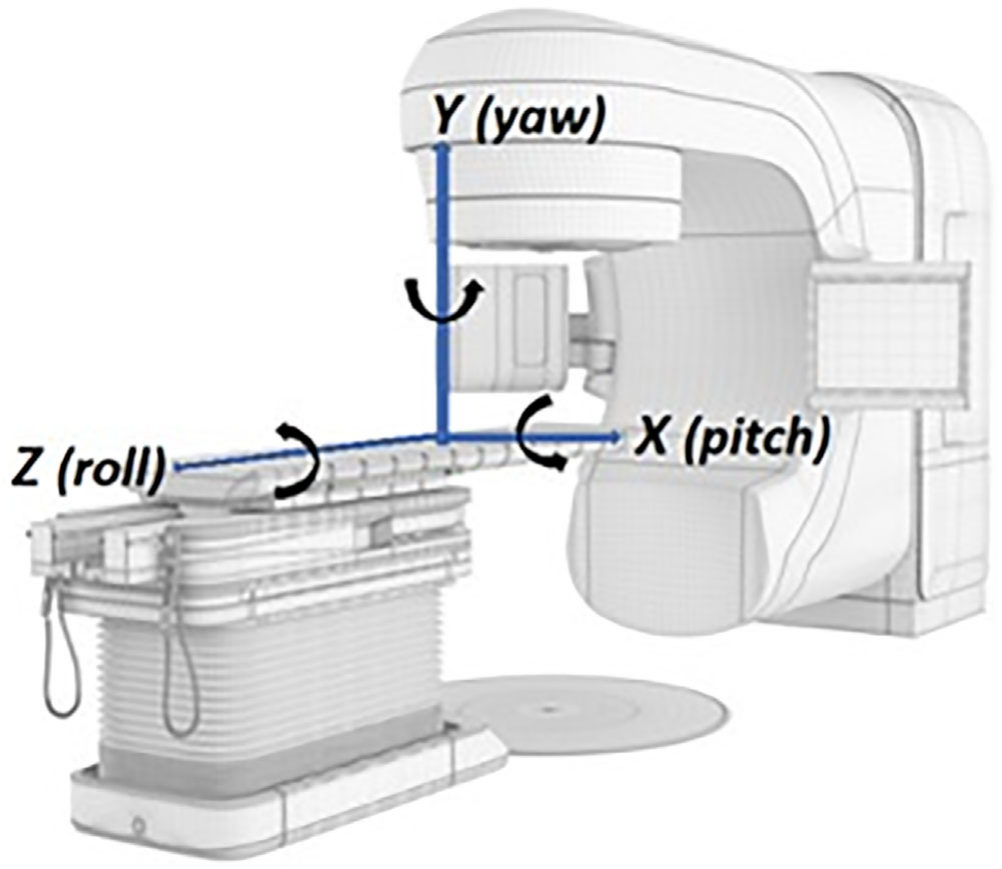
Definition of pitch, roll, and yaw

The dosimetric effect of patient rotation was evaluated by simulating multiple rotational combinations in the Eclipse treatment planning system (TPS) using the AAA algorithm. Since the commercial TPS will not perform calculations on rotated CT image sets with skewed pixels, we used Velocity AI (Varian Medical Systems, Palo Alto, CA) to simulate patient rotations. To do this, we truncated the planning CT exactly 10 cm above and below the isocenter, such that the resultant 3D Image “tilt 0” is centered at the isocenter level in the superior/inferior direction. This image set in Velocity was rotated around the pivot point of the image center in 12 different rotational combinations and defined as 1A‐3D in Table 1. The definition of pitch, roll and yaw is illustrated in Figure 2.

**TABLE 1:** Twelve different rotational combinations

	1A	1B	1C	1D	2A	2B	2C	2D	3A	3B	3C	3D
Pitch x	−1°	−1°	+1°	+1°	−2°	−2°	+2°	+2°	−3°	−3°	+3°	+3°
Yaw y	−1°	+1°	−1°	+1°	−2°	+2°	−2°	+2°	−3°	+3°	−3°	+3°

Rotational error in roll was not considered in this study, as both the original TB plans and the recreated Halcyon plans were coplanar VMAT with three to four full arcs, and a 1–3° patient roll would not be significant compared to several 360° arcs; each one degree of roll will contribute an error of 1/360, and if a plan has 3 arcs, the contribution will be < 0.1%. This was validated by a pilot study.

**FIGURE 3:**
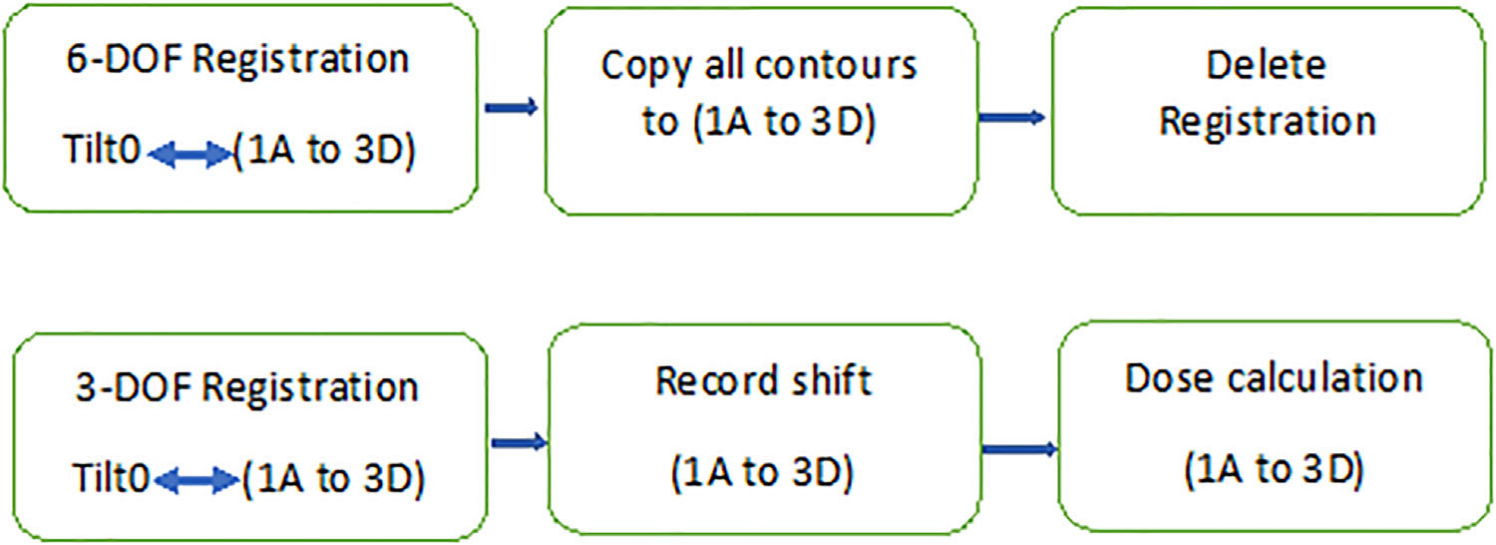
6‐DOF and 3‐DOF registration flow chart

The twelve rotated images were imported back to Eclipse, followed by a series of 6‐DOF chain registration with auto‐matching (Figure 3) between “tilt 0” and 1A 1B 1C 1D 2A 2B 2C 2D 3A 3B 3C 3D. Finally, all contours were overlaid with the fused rotated images after double checking the rotation matrix at technical properties with X = 1° for 1° pitch, Y = 1° for 1° yaw, and so forth.

We did not rotate the contours in Velocity due to volume variation after multiple manipulations followed by image resampling. Instead, we copied the original structures to registered image after a 6‐DOF auto matching in Eclipse. We validated the fidelity of the overlaid contours by calculating the original beam on each perfectly registered (6‐DOF) CT image set and comparing the DVH to that of the original plan, which were identical.

**FIGURE 4:**
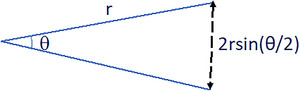
Rotational error projection at distance

After confirming the integrity of the contour and rotation, we deleted all the 6‐DOF registration, we then performed a new set of 3‐DOF chain registrations with translation alone and recorded the translational shift for correcting each rotational combination (Figure 4). This could mimic the clinical situation where no rotational correction, and only translational correction is available, as in the Halcyon treatment scenario. Dose was recalculated with the same MU on the CT image set with both TB and Halcyon plans. This resulted in 24 sets of treatment plans for each patient, with 8 subsets per 1°, 2° and 3° rotated images respectively.

### 2.3 Plan evaluation

All plans are evaluated pairwise (paired 2‐sided *t*‐test) using dose‐volume histograms (DVHs). PTV coverage was evaluated using the Conformity Index (CI), Gradient Index (GI) and the Homogeneity index (HI) as follows: 

RTOG Conformity Index: CI = PIV/TV; PIV is the prescription isodose volume; TV is the target volume.

Paddick Conformity Index:  pCI = TVPV^2^ / (TV × PIV); where PIV is the prescription isodose volume, TVPV is the target volume within the prescribed isodose surface, and TV is the target volume.

Paddick Gradient Index: pGI = PhV/PIV; where PhV is the volume of half the prescription isodose; PIV is the volume of the prescription isodose line. For a plan prescribed to the 80% isodose line, it is the ratio of the 40% isodose volume to that of the 80% isodose volume.

RTOG Homogeneity Index: HI = *D*_max_/*D*_p_; where *D*max is the maximum point dose and *D*_p_ is the prescribed dose to the target volume.

To quantitatively measure the PTV coverage and OAR sparing of each plan, an in‐house Eclipse script was developed to extract data from the DVH. We compared PTV coverage, CI, pCI, pGI, and HI.

To compare OAR sparing, we collected the V8Gy, V10Gy, and V12Gy, as well as the D0.03cc to represent maximum OAR dose. The total monitor units (MUs) per fraction and MLC segments were compared to estimate the plan complexity for each plan.

### 2.4 Statistical analysis

Data were tabulated as mean ± standard deviation. Statistical analysis was performed utilizing Microsoft Excel for paired two‐sided *t*‐test. For plan quality, the Halcyon plan was compared with TB plan. For delivery accuracy, the translation corrected plan was compared to the perfect‐setup plans in both TB and Halcyon. A value of *p* = 0.05 or below was statistically significant.

## 3 RESULTS

Rotational error in roll was not considered in this study, we assumed if a plan has three arcs, the contribution will be < 0.1%. This was validated by a pilot study. Results showed the dosimetric influence of patient rotational setup error was statistically insignificant for rolls of 2° (Table 2).

**TABLE 2:** Comparison of dosimetric indices of roll 2 and P1 R1 Y1.5 on Halcyon

Variable	Roll 0	Roll 2	P1 R1 Y1.5
RTOG Conformity Index (CI)	1.12 ± 0.33	1.12 ± 0.33	1.12 ± 0.33
Paddick Conformity Index (pCI)	0.78 ± 0.12	0.77 ± 0.12	0.77± 0.12
Paddick Gradient Index (pGI)	4.08 ± 0.84	4.07 ± 0.84	4.08 ± 0.84
RTOG Homogeneity index (HI)	1.33 ± 0.10	1.34 ± 0.10	1.33 ± 0.10
PTV Coverage (%)	90.8± 5.2	90.7 ± 4.6	90.7 ± 4.6
Max cord/cauda (Gy)	11.9±2.6	12.2±2.4	11.8±2.4
Spinal cord/cauda V8 (cc)	2.2±2.2	2.2±2.2	2.2±2.1
Spinal cord/cauda V10 (cc)	1.5±2.2	1.2±1.8	1.1±1.8
Spinal cord/cauda V12 (cc)	1.1±2.0	0.8±1.5	0.7±1.5

*Note*: All *p*‐values > 0.05 (n = 20, paired *t*‐test, 2 tails). Roll 0 = 0° roll; Roll 2 = 2° roll; *P1 R1 Y1.5 *= 1° pitch, 1° roll, 1.5° yaw.

Analysis of the 50 cone beam computed tomography (CBCT) scans showed average rotational setup errors of 0.6°, 0.8°, and 1.2° in pitch, roll, and yaw, respectively. So, we analyzed rotational setup errors of 1°, 1°, and 1.5° in pitch, roll, and yaw respectively. Results showed the dosimetric influence was not statistically significant (Table 2).

At the time of registering rotated images, we recorded the translational shift for correcting each rotational combination (Table 3). Our results showed that without rotational correction, the average absolute translations were 0.2, 0.3, and 0.9 mm in X, Y, and Z, respectively for 1° pitch/yaw combinations, 0.4, 0.4, and 1.7 mm for X, Y, and Z, respectively for 2° pitch/yaw combinations, and 0.5, 0.5, and 2.6 mm for X, Y, and Z, respectively for 3° random pitch/yaw combinations.

**TABLE 3:** Average translational shift for correcting each rotational combination

Rotation	X (mm)	Y (mm)	Z (mm)
1°	0.23±0.38	0.28±0.35	0.94±0.60
2°	0.38±0.52	0.38±0.47	1.72±0.95
3°	0.45±0.70	0.50±0.73	2.55±1.35

Table 4 summarizes the plan quality of TB versus Halcyon. We find that with the same number of arcs, Halcyon plans are comparable to TB with similar target coverage and max cord/cauda dose. However, the average target coverage was about 1.2% lower. Although this was not statistically significant (*p* = 0.24), it is considered clinically unacceptable. After adding one additional arc, we achieved acceptable plans reviewed by physicians with similar target coverage (92.3 ± 3.0% vs. 92.4 ± 3.3%, *p* = 0.82), similar pCI (0.83 ± 0.1 vs. 0.81 ± 0.1, *p* = 0.17), and similar HI (1.31 ± 0.07 vs. 1.31 ± 0.08, *p* = 0.84) compared to TB plans. Halcyon plans have higher Gradient Index (3.96 ±0.8) than TB plans (3.85 ±0.7) without statistical significance (*p* = 0.21). Maximum dose to the spinal cord/cauda was also comparable (11.1 ± 2.8 Gy vs. 11.4 ± 3.6 Gy, *p* = 0.39), as were the V8Gy, V10Gy, and V12Gy for the cord/cauda. The total MUs for Halcyon (4998 ± 1688) were comparable to TB (5463 ± 2155).

**TABLE 4:** Comparison of dosimetric indices of Halcyon versus TB

Variable	TB 2–3 VMAT	HAL 2–3VMAT	HAL 3–4VMAT
RTOG Conformity Index (CI)	1.00 ± 0.1	1.10 ± 0.4	1.10 ± 0.2
Paddick Conformity Index (pCI)	0.83 ± 0.1	0.77 ± 0.1*	0.81 ± 0.1
Paddick Gradient Index (pGI)	3.85 ± 0.7	4.06 ± 0.9	3.96± 0.8
RTOG Homogeneity index (HI)	1.31 ± 0.1	1.34 ± 0.1	1.31 ± 0.1
Total MU	5463±2155	4775±1591	4998±1688
PTV coverage (%)	92.30 ± 3.0	91.10 ± 5.4	92.40 ± 3.3
Max cord/cauda (Gy)	11.10±2.8	11.20±2.5	11.40±3.6
Spinal cord/cauda V8 (cc)	2.10±2.2	2.30±2.1	2.30±1.8
Spinal cord/cauda V10 (cc)	1.10±1.8	1.20±1.9	1.00±1.6
Spinal cord/cauda V12 (cc)	0.60±1.3	0.80±1.5	0.60±1.4

*Note*: All *p*‐values > 0.05 (*n* = 20, paired *t*‐test, 2 tails) *except* ^*^ *p*≤0.05.

We further quantified dosimetric effect of correcting rotation with translation alone on TB (Table 5) and Halcyon (Table 6). In both TB and Halcyon, the dosimetric influence of patient rotational setup error was statistically insignificant for rotations of up to 1° pitch/yaw if corrected by couch translation (with similar target coverage, CI, max cord/cauda dose and V8Gy, V10Gy, V12Gy for cord/cauda). At combined rotations of 2° pitch/yaw, the dosimetric influence of patient rotational setup error was statistically significant for TB, but not significant for Halcyon: target coverage decreased by 0.6% for Halcyon and 1% for TB. Target coverage did not go down significantly for Halcyon until combined rotations of 3°, with an average 1.7% drop of target coverage. Despite a statistically significant drop in target coverage, the cord/cauda maximum dose and V8Gy‐V12Gy were comparable.

**TABLE 5:** Comparison of dosimetric indices on 1°–2° rotated image on TB

Variable	Tilt 0	Rotation	A	B	C	D
Conformity Index (CI)	1.04±0.12	1°	1.04±0.12	1.04±0.12	1.04±0.12	1.04±0.12
		2°	1.04±0.12	1.04±0.13	1.04±0.12	1.04±0.12
PTV Coverage (%)	92.3±3.05	1°	92.00±3.02	92.00±3.14	92.20±2.85	92.20±3.00
		2°	91.30±3.36*	91.50±3.51*	91.30±2.50*	91.30±2.97*
Max cord/cauda (Gy)	10.4±1.80	1°	10.40±1.84	10.50±1.90	10.40±1.75	10.50±1.85
		2°	10.50±1.91	10.60±2.02	10.80±1.76	10.70±2.02
Spinal cord/cauda V8 (cc)	1.77±1.76	1°	1.70±1.77	1.71±1.75	1.72±1.77	1.74±1.75
		2°	1.70±1.77	1.70±1.74	1.74±1.74	1.70±1.72
Spinal cord/cauda V10 (cc)	0.69±1.43	1°	0.67±1.41	0.67±1.38	0.68±1.40	0.68±1.38
		2°	0.68±1.40	0.68±1.38	0.70±1.38	0.69±1.39
Spinal cord/cauda V12 (cc)	0.26±0.79	1°	0.27±0.79	0.26±0.76	0.26±0.77	0.25±0.75
		2°	0.28±0.80	0.27±0.78	0.27±0.76	0.27±0.77

*Note*: *n = *20, paired *t*‐test, 2 tails; all *p‐value >* 0.0*5* except ^*^p≤0.05. A = (−1°–2° pitch/−1°–2° yaw), *B* = (−1°–2° pitch/+1°–2° yaw), *C* = (+1°–2° pitch/−1°–2° yaw), *D* = (+1°–2° pitch/+1–2° yaw).

**TABLE 6:** Comparison of Dosimetric indices on 1°–3° rotated image on Halcyon

Variable	Tilt 0	Rotation	A	B	C	D
Conformity Index (CI)	1.09±0.24	1°	1.10±0.24	1.10±0.24	1.10±0.24	1.10±0.24
		2°	1.10±0.24	1.10±0.25	1.10±0.24	1.10±0.24
		3°	1.09±0.25	1.09±0.25	1.09±0.24	1.09±0.24
PTV Coverage (%)	91.1±5.35	1°	91.00±5.55	91.00±5.57	91.20±5.37	91.10±5.64
		2°	90.60±5.88	90.50±5.93	90.70±5.52	90.40±5.82
		3°	89.30±5.86*	89.20±6.12*	89.90±5.40*	89.20±5.81*
Max Cord/cauda (Gy)	11.2±2.47	1°	11.20±2.48	11.30±2.49	11.20±2.49	11.30±2.49
		2°	11.20±2.55	11.30±2.58	11.40±2.48	11.40±2.51
		3°	11.40±2.61	11.50±2.65	11.50±2.34	11.50±2.58
Spinal Cord/cauda V8 (cc)	2.34±2.14	1°	2.29±2.12	2.30±2.13	2.31±2.12	2.30±2.13
		2°	2.28±2.11	2.28±2.13	2.31±2.10	2.28±2.09
		3°	2.27±2.12	2.25±2.12	2.29±2.06	2.28±2.08
Spinal Cord/cauda V10 (cc)	1.19±1.86	1°	1.22±1.86	1.23±1.86	1.22±1.82	1.22±1.82
		2°	1.21±1.87	1.23±1.86	1.22±1.83	1.21±1.84
		3°	1.20±1.82	1.17±1.80	1.18±1.78	1.17±1.77
Spinal Cord/cauda V12 (cc)	0.75±1.54	1°	0.76±1.48	0.75±1.46	0.76±1.51	0.74±1.48
		2°	0.77±1.49	0.76±1.47	0.76±1.49	0.78±1.51
		3°	0.74±1.49	0.71±1.44	0.76±1.51	0.72±1.46

*Note*: *n = *20, paired *t*‐test, 2 tails; all *p*‐values* >* 0.05 except ^*^p≤0.05. A = (−1°–3° pitch/−1°–3° yaw), *B* = (−1°–3° pitch/+1–3°yaw); *C* = (+1°–3° pitch/−1°–3° yaw), *D* = (+1–3°pitch/+1–3°yaw).

In Patients #5 and #12, there is no gap between the target and cord/cauda. After creating a 2 mm gap, the maximum cord dose difference was reduced from 2.38 Gy to 0.2 Gy for the 2C rotation in patient #5, and from 2.77 Gy down to 0.8 Gy for patient #12 on 2D rotation (Table 7). This also confirms that a minimum distance between the target and cord/cauda OAR is needed for Halcyon SBRT Delivery.

**TABLE 7:** Comparison of max cord dose difference (Gy) of 2° rotated image on Halcyon and TB

	Halcyon				TB			
Patients	2A	2B	2C	2D	2A	2B	2C	2D
1	0.00	−0.01	−0.02	−0.01	0.1	0.02	0.06	0.03
2	−0.08	−0.10	−0.04	−0.05	0.01	0.04	−0.03	−0.05
3	0.45	−0.07	0.40	−0.06	0.82	−0.26	1.36	0.27
4	0.10	0.04	0.36	0.24	0.08	0.04	0.34	0.31
5	−0.11	0.23	2.38	−0.55	−0.12	0.32	2.90	−0.49
6	−0.29	−0.08	0.35	0.25	0.17	0.11	0.52	−0.03
7	0.45	0.54	0.86	1.11	0.34	0.43	0.34	0.63
8	−0.02	0.00	0.00	0.00	−0.02	−0.03	−0.05	−0.04
9	0.52	0.65	−0.87	−0.66	0.88	1.28	−1.13	−0.9
10	−0.02	0.02	−0.06	−0.07	0.48	0.35	0.88	0.86
11	0.16	−0.18	0.21	−0.42	0.23	0	0.53	0.02
12	−1.17	1.01	−0.69	2.77	−1.84	1.52	−1.01	3.17
13	0.15	0.04	0.21	0.70	0.18	0.35	0.42	0.98
14	−0.02	−0.12	0.47	0.00	0.12	−0.34	0.28	−0.19
15	0.09	−0.12	0.13	0.09	0.22	0	0.21	0.17
16	−0.31	−0.58	−0.02	−0.47	−0.2	−0.3	−0.04	−0.17
17	0.08	−0.01	−0.02	−0.04	0.14	0.06	−0.07	−0.11
18	0.06	0.13	−0.05	−0.09	0.08	0.13	0.12	0.07
19	0.00	−0.04	0.09	0.08	0.29	−0.03	0.27	0.17
20	−0.06	−0.12	0.15	0.12	−0.13	−0.19	0.23	0.21

## 4 DISCUSSION

With one full arc added to the original VMAT plan, Halcyon plans for a single spine metastasis were dosimetrically comparable to TB plans. Previous study by Heather^21^ et al. also demonstrated that the Halcyon™ dual‐layer MLC can generate comparable and clinically equivalent spine SBRT plans to TrueBeam plans. If the setup error in patient rotation is within 1° in the pitch and yaw directions, translational corrections can be applied such that the dosimetric effect was insignificant for both Halcyon and TB. This demonstrates that it is possible to treat such patients on Halcyon, without a compromise in accuracy of delivery.  If the rotational error reaches up to 2°, the dosimetric influence of patient rotational setup error can still be corrected for by couch translation for Halcyon plans. As TB MLCs have finer leaves (2.5 mm leaf width is an optional purchase), TB plans achieve a sharper dose fall off when compared to Halcyon plans. TB plans may be more sensitive to rotational errors due to the higher gradient between the target edge and the spinal cord/cauda, which can partially explain the deterioration of plan quality at 2° rotations corrected by translation.

All retro‐generated Halcyon plans were reviewed by our physician and meet institutional constraints. We then scaled all prescription doses to 16 Gy in 1 fraction in order to average the maximum cord/cauda dose from the plans.

The standard deviation was large for OARs, likely due to 2 factors: different dose limits for spinal cord/cauda equina as well as rescaling of the original prescription from 12–16 Gy in 1 fraction or 18–24 Gy in 3 fractions to 16 Gy in 1 fraction, which resulted in larger OAR dose variation and hence a larger standard deviation. However, since we used paired *t*‐test for this case‐control study, plans from same patient were compared with and without rotational error. Thus, the larger standard deviation would not invalidate these statistics.

We compared un‐rotated plans with plans rotated up to 2° and corrected by translation. Overall, neither max cord/cauda dose nor V8Gy‐V12Gy cord/cauda volume demonstrated any statistical significance. We attribute this to two factors: first is there is a minimum of 2 mm gap between the target and cord/cauda in most patients, second is the isocenter is close to the center of the cord/cauda in majority of the cases, which makes it less sensitive to rotational setup errors. However, after examining each individual patient, we found in Patient #5 at 2C rotation, the maximum cord/cauda dose increased by 3.57 Gy and in patient #12 at 2D rotation, the maximum cord/cauda dose increased by 2.09 Gy (Table 7). These increases in OAR dose would be considered clinically unacceptable.

After further examination of the contours, we found that in these two patients, the target and OAR had no separation on certain slices. This was different than other cases, where a 2 mm separation typically exists between the target and the cord/cauda. Although patients #3 and #9 also had no separation between target and OAR, the dose to cord/cauda did not differ significantly with rotation. To evaluate this, we measured the axial distance from center of spinal cord/cauda to isocenter (placed at the center of the target). We noticed in case #3 and #9, the isocenter to cord distance was small, but in case #5 and #12, the isocenter to cord distance was much larger (Table 8).

**TABLE 8:** Isocenter to cord distance

Patients	AP (mm)	L/R (mm)
1	25.4	1.5
2	16	5.7
3	17	10
4	28	11
5	0	29
6	24	2
7	25	1
8	19	9
9	0	1
10	1	2
11	28	1
12	22	41
13	9	1
14	8	30
15	25	0
16	33	3
17	24	0
18	25	3
19	22	3
20	24	2

We had the following assumption: if the isocenter is away from the cord/cauda by a distance of “r”, then for a *θ*° rotation, at least (2rsin (*θ*/2)) margin to the cord/cauda is needed (Figure 4). For example, if an isocenter is 3 cm away from the cord/cauda, then for any 2° rotation, at least 1 mm margin to the cord/cauda is needed (Table 9). For an isocenter 4 cm away from the cord/cauda, then for any 2° rotation, at least 1.4 mm margin to the cord/cauda is needed. In patients #5 and #9, the isocenter were 3 and 4 cm away from the cord/cauda. Therefore, for a 2° rotational set up error, a 1 and 1.4 mm margin to the cord/cauda would be needed. The distance between spinal cord/cauda and isocenter were much smaller in patients #3 and #9, thus no significant maximum cord/cauda dose difference.

**TABLE 9:** Safety margin for cord/cauda without rotational correction

Iso to cord distance (mm)	Patient rotation (°)	Cord margin needed (mm)
10	2	0.3
20	2	0.7
30	2	1.0
40	2	1.4
50	2	1.7
10	3	0.5
20	3	1.0
30	3	1.6
40	3	2.1
50	3	2.6

To verify our assumptions, we intentionally created a gap between the target and cord/cauda on patients #5 and #12, and found that for patient #5, the maximum OAR dose difference was reduced from 3.57 Gy to 0.2 Gy for the 2C rotation, and from 2.09 Gy down to 0.8 Gy for patient #9 on 2D rotation in Halcyon. This also confirms that a minimum distance between the target and cord/cauda OAR is needed for Halcyon SBRT Delivery. So, for safe implementation of SBRT on Halcyon for a tumor in a single thoracic or lumbar vertebral segment, a safety gap between the target and spinal cord/cauda of at least 2 mm is necessary.

Studies have shown that for most cases, rotational correction is not as essential when compared to translational errors. Hyde^7^ et al. reported that the translational and rotational errors in patient positioning were relatively small. From a cohort of 307 image registrations for spinal metastasis treatment, 90% of translational positioning errors were within 1 mm and 97% of rotational positioning errors were within 1°. For most rotational positioning errors, compensation is not needed for the 1° error. Translational positioning errors always need to be addressed because it poses dosimetric risk to nearby critical organs. Extending the treatment volume to two or more vertebral levels would amplify the translational movement, and therefore positional error, at the cephalad and caudal borders of the target volume and therefore should be avoided when rotational corrections in all planes cannot be easily achieved. 

Our study showed that if we minimize the distance between the isocenter and Cord/Cauda by placing the isocenter inside the target toward the Cord/cauda direction, and also maintain a minimum distance of 2 mm between the target and cord/cauda, Halcyon treatment of single thoracic or lumbar vertebral segment tumor is feasible.

## 5 CONCLUSION

Halcyon treatment of single thoracic or lumbar vertebral segment tumor is feasible, but caution should be taken with patients requiring rotational corrections of > 1° in the absence of 6‐DOF correction capabilities. It is advisable to choose the patients appropriately, including only those with thoracic or lumbar spine involvement and keeping at least 2 mm separation between the target and the cord/cauda. More margin is needed if the distance between the isocenter and cord/cauda are larger, and frequent imaging to monitor the patient will also be helpful. It is also advisable to place the planning isocenter close to the spinal canal to further mitigate the rotational error.

## CONFLICT OF INTEREST

The authors declare no conflict of interest.

## AUTHOR CONTRIBUTIONS

Jeonghoon Park developed the in‐house Eclipse script to extract data from the DVH, Ron Lalonde and Saiful Huq helped with study design, Si Young Jang and Maria Stefania diMayorca did all the VMAT Plan QA, John Flickinger and Andrew Keller reviewed the plan. All coauthors reviewed and edited the manuscript.

## Data Availability

All data generated and analyzed during this study are included in this published article (and its Supporting Information files).
